# World AIDS Day: together we will stop HIV transmission and conquer AIDS

**DOI:** 10.1186/1742-4690-10-129

**Published:** 2013-11-15

**Authors:** Mark A Wainberg, Andrew Lever

**Affiliations:** 1 McGill University AIDS Centre, Lady Davis Institute, Jewish General Hospital, Montreal Québec, Canada; 2 Department of Medicine, University of Cambridge, Addenbrooke's Hospital, Cambridge CB2 0QQ, UK

## 

It is fair to say that the HIV/AIDS epidemic is unprecedented for a number of different reasons. As another World AIDS Day approaches on December 1, it behooves us to take stock of the progress that has been made and reflect on the road that still lies ahead.

First, who can remember another disease that arose as quickly as HIV did in the early 1980s to infect millions of people around the world, almost all of whom succumbed to their illnesses at a time that antiretroviral (ARV) drugs were non-available. Second, the development of safe and well-tolerated ARVs over the past 25 years has now resulted in a situation in which almost all infected persons, who are fortunate to live in wealthy countries, can aspire to live for many years, as HIV disease has been transformed into a chronic manageable condition. Of course, many problems remain, not the least of which is that HIV continues to spread to millions more people each year. In addition, people who live in developing countries are often treated with inferior drugs compared to those that are now available in wealthier settings and are, therefore, less likely to fully benefit from the treatment advances that have taken place.

Against this background, there is now a widespread consensus that the only truly effective way to deal with the HIV epidemic over the long term will be to find a cure. First, although the global programmes that exist to provide ARVs to people in developing countries (who could not otherwise afford them) have been successful, they may well be unsustainable over the long term for reasons of cost. Simply put, the total costs may well exceed hundreds of billions of dollars over the next decade and many health care economists have sounded the alarm that the West may not be able to provide this necessary assistance unless the worldwide economy improves. Second, it is no panacea for anyone to need to take drugs every day for the rest of their life. And, so far at least, the quest for an effective HIV vaccine has fallen flat, in spite of valiant and insightful efforts by scientists throughout the world.

In contrast, the optimism that now exists in regard to potential curative strategies is palpable [1,2]. Among other considerations, we now possess a much fuller understanding of the problems involved and recognize that HIV has been able to establish itself in latent form in long-lived cellular reservoirs that cannot be easily targeted by currently available ARVs [3]. This has resulted in a large number of novel concepts that are aimed at the reactivation of these reservoirs, so that latently infected cells may be effectively targeted by more traditional drugs [4]. In almost all western countries, both public and private granting agencies have now established dedicated funding programmes that seek to attain a cure for HIV. And, for the first time, a large critical mass of scientists are fully engaged in this effort. Indeed, the goal of finding a cure for HIV was highlighted at the recent Frontiers in Retrovirology conference in Cambridge, United Kingdom, where more than 300 scientists converged to discuss this topic. These individuals are working on a large variety of approaches that share this same goal. Hopefully, at least some of these efforts will be successful.

It is also important to point out that research is flourishing in a number of related areas as well. For example, the success of current ARV usage in the treatment of HIV has provided benefits to both society as a whole as well as to individuals. We now know that people who are successfully treated have vastly diminished viral loads in their bodies and, as a consequence, are far less able to transmit HIV than would otherwise happen [5,6]. On a population level, it has been shown that the use of ARVs has led to significant reductions in community viral load, which refers to the average viral burden in a community of HIV- infected individuals.

These observations have given rise to efforts to both protect against new infections through the use of ARVs administered on a prophylactic basis to people who might be at risk for acquisition of HIV in programmes that are referred to as pre-exposure prophylaxis (PrEP) [7]. To date, certain of these studies suggest that PrEP may be able to protect as many as 50% of individuals at risk from acquisition of HIV, so long as they take their ARVs as prescribed.

There has also been stimulation of a related area of research termed Treatment as Prevention (TasP) that holds that the successful mass use of ARVs will lead to diminished viral loads across populations, such that the transmission of new HIV infections will be greatly reduced or halted [8]. Although concerns have often been expressed that the development of HIV drug resistance and the transmission of drug-resistant viruses might thwart such efforts, the recent development of novel compounds that may not be as prone to drug resistance as earlier drugs may help to provide a solution to this problem.

We have more of a right to be optimistic today than at any time since the outbreak of the HIV epidemic. Hopefully, we are now closer to the end than to the beginning of the HIV epidemic and the observation of World AIDS Day will ultimately become a cause for celebration of our cumulative success.

## Authors’ information

Mark A Wainberg and Andrew Lever are the co-Editors-in-Chief of Retrovirology.
